# Epidemiological Analysis of Cardiovascular Diseases with Consideration of Risk Factors, Health Awareness, and Preventive Behaviors in Civilian and Military Populations

**DOI:** 10.3390/jcm14165844

**Published:** 2025-08-18

**Authors:** Magdalena Zawadzka, Ewelina Ejchman-Pac, Amelia Kowalska, Paweł Szymański, Justyna Marszałkowska-Jakubik

**Affiliations:** 1 Department of Health Education, Prevention, and Promotion, Military Institute of Hygiene and Epidemiology, Kozielska Street 4, 01-163 Warsaw, Poland; magdalena.zawadzka@umed.lodz.pl (M.Z.); ewelina.e.pac@wihe.pl (E.E.-P.); 2 Department of Epidemiology and Public Health, Medical University of Lodz, Żeligowskiego Street 7/9, 90-752 Lodz, Poland; amelia.kowalska@student.umed.lodz.pl; 3 Department of Radiobiology and Radiation Protection, Military Institute of Hygiene and Epidemiology, Kozielska Street 4, 01-163 Warsaw, Poland; pawel.szymanski@umed.lodz.pl; 4 Department of Pharmaceutical Chemistry, Drug Analysis and Radiopharmacy, Medical University of Lodz, Muszyńskiego Street 1, 90-151 Lodz, Poland

**Keywords:** cardiovascular disease risk factors, military and civilian populations, health awareness, preventive behaviors, epidemiological study

## Abstract

**Background**: Cardiovascular diseases (CVD) remain the leading cause of death in both Poland and worldwide. Despite a decline in CVD-related mortality observed in Poland since 1991, national rates still exceed the European Union average. **Methods**: The aim of this study was to assess the prevalence of cardiovascular risk factors and the level of health awareness among Polish Armed Forces personnel, including both soldiers and civilian employees. A total of 308 participants (82.00% soldiers) underwent anthropometric measurements, lipid profile testing, and completed a structured questionnaire. **Results**: The results indicate a high prevalence of modifiable risk factors, such as overweight (30.30%), low physical activity (21.20%), and tobacco use (21.20%). Additionally, 54.00% of respondents reported experiencing stress, and 17.00% had elevated cholesterol levels. Statistically significant associations were found between selected parameters and gender, age, service corps, and occupational status. Despite a moderate level of knowledge regarding CVD, the majority of participants expressed a willingness to expand their understanding. **Conclusions:** The findings highlight the importance of early prevention and health education on cardiovascular diseases, especially in military environments where stress and lifestyle factors may contribute to increased risk.

## 1. Introduction

Since 1991, Poland has observed a consistent decline in mortality due to cardiovascular diseases (CVD). A similar trend has been noted in the incidence of ischemic heart disease, particularly among men. In 1995, morbidity rates in men were markedly higher compared to those in women; however, by 2017, the rates had reached comparable levels [1]. Despite these improvements, CVD-related mortality rates in Poland and other Central and Eastern European (CEE) countries remain significantly higher than the European Union (EU) average. Residents of Poland have a shorter life expectancy—by approximately 3 to 7 years—compared to citizens of the “old” EU member states. A key determinant of this disparity is the higher CVD mortality among Polish men and women relative to the majority of other EU countries [2,3,4].

Globally, cardiovascular diseases are the leading cause of death, accounting for approximately one-third of all deaths worldwide [5]. In Poland, as of 2023, cardiovascular diseases were responsible for 40.00% of all deaths, translating to 40 CVD-related deaths per 10,000 inhabitants [6]. This figure was even higher in 2010, reaching 46% [4]. Not only are CVD mortality rates higher in CEE countries, but cardiovascular events also tend to occur at a younger age. In some CEE countries—such as Belarus, Kazakhstan, Kyrgyzstan, Russia, and Ukraine—CVD mortality rates among men aged 55–59 years are higher than those observed among men aged 75–79 years in France [2].

Current efforts remain insufficient to address the scale of the CVD epidemic in the CEE region. Strengthening the epidemiological data on cardiovascular health in this area is critical to support surveillance and progress in the implementation of CVD prevention strategies, particularly in the most affected populations [2]. There is no universal solution for improving cardiovascular health globally. However, advances in cardiology over the past 50 years have provided tools and knowledge that allow for significant mitigation of CVD-related harm [5]. In the military population, the prevalence of hypertension is particularly concerning. In Gielerak’s study, hypertension was diagnosed in 44.70% of soldiers over the age of 50, with more than half showing elevated values in office measurements, and 14% meeting the criteria for stages 2 or 3 hypertension. Among soldiers with normal blood pressure, one-third had borderline values, and 86.00% of those with a history of hypertension had elevated readings during the examination [7]. Even higher rates were reported among candidates for service in the Navy—cardiovascular diseases were the cause of disqualification in 243 individuals, accounting for 4.60% of all those deemed unfit and 1.20% of all examined; the most frequent diagnosis was hypertension (32.50% of all cardiovascular conditions) [8]. For comparison, according to 2023 data from the Polish Ministry of Health, hypertension affects 32% of adult Poles, with this proportion rising to 75.00% among those aged 65 and older [9]. This suggests that the military population—despite theoretically better initial health—becomes exposed over time to cardiovascular risks equal to or even greater than those in the general population. These findings reinforce the need for early and systematic prevention, particularly in light of the occupational burdens specific to military service. The decline in mortality from ischemic heart disease observed in Poland has been attributed primarily to lifestyle changes and reductions in risk factors among the adult population. Therefore, understanding the prevalence of cardiovascular risk factors is essential to inform and guide preventive efforts [4].

Non-modifiable risk factors for CVD include age, sex, ethnicity, and family history (often associated with polymorphisms in specific genes).

Modifiable risk factors can be categorized into two groups:

1. Metabolic factors: systolic blood pressure, low-density lipoprotein (LDL) cholesterol levels, body mass index (BMI), fasting blood glucose, and kidney function impairment;
2. Behavioral (lifestyle-related) factors: diet, smoking, exposure to secondhand smoke, alcohol consumption, and physical activity.

Environmental factors—such as outdoor air pollution, household air pollution, lead exposure, and extreme temperatures—are considered partially modifiable risk factors [10].

Tobacco use is one of the principal modifiable risk factors for cardiovascular diseases, alongside hypertension, diabetes, obesity, and elevated LDL cholesterol levels. Smokers are at twice the risk of cardiovascular mortality compared to individuals who have never smoked [8]. National tobacco control programs—implemented in 91% of countries—are the most widespread global public health intervention in this domain [5]. Worldwide, tobacco use prevalence declined from 32.70% in 2000 to 22.30% in 2020 [11]. In military populations, tobacco use is more prevalent than in civilian settings, which may be associated with cultural norms and occupational stress.

A similarly concerning behavioral risk factor is alcohol misuse, which is particularly prevalent among military personnel. Serving in the armed forces is associated with high levels of psychological stress and exposure to traumatic experiences, which, in some cases, can lead to harmful drinking patterns. Studies confirm that alcohol consumption is more common among soldiers and veterans than in the civilian population [12,13,14,15]. Woodruff et al. (2010) also emphasize the role of easy access to alcohol on military bases, reduced prices, limited recreational opportunities, and the cultural acceptance of drinking within the military environment. Social stigma and fear of negative career consequences further discourage individuals from seeking therapeutic support [16]. As a result, alcohol misuse in the armed forces represents a complex and serious public health challenge, negatively affecting operational readiness, health status, and the long-term functioning of service members.

Given the unique challenges of military service, early intervention and sustained prevention are key to reducing cardiovascular burden.

## 2. Materials and Methods

The consent to conduct this study was obtained from the Bioethics Committee of the Military Medical Commission in Warsaw (Resolution No. 11/23). This study was conducted in accordance with the Declaration of Helsinki of the World Medical Association. The study population was selected in accordance with the internal procedures of the government institution. Convenience sampling was used in this study. The research was conducted in a total of seven military units. Due to applicable data protection regulations, the detailed criteria for sample selection remain undisclosed. Each subject was informed of the details of this study, its purpose, and the benefits of participation. All participants gave written, informed consent to participate in this study and completed an original questionnaire assessing the knowledge of soldiers/civilian military employees about cardiovascular diseases and the risk factors for their occurrence.

Study Design:

This study consisted of the following stages:

1. Eligibility for participation in this study, based on inclusion and exclusion criteria;
2. Expressing written, informed consent to participate in this study;
3. Survey study;
4. Blood pressure measurement;
5. Blood sampling for cardiovascular risk parameters;
6. Laboratory testing—lipid profile parameters (total cholesterol, HDL cholesterol, LDL cholesterol, triglycerides) in the context of cardiovascular risk;
7. Entering the obtained results into the database;
8. Statistical analysis of the obtained results.

Inclusion Criteria:

1. Soldier or civilian employee of the military;
2. Provision of informed consent to participate in this study.

Exclusion Criteria:

1. Lack of informed consent to participate in this study;
2. Any clinical condition that prevents cooperation;
3. Cancer or acute chronic inflammatory conditions.

The data on the presence or absence of cardiovascular or other chronic diseases are based on participants’ self-reported responses.

Blood pressure was measured by qualified medical personnel using an OMRON M7 Intelli IT device. Body mass index (BMI) was calculated based on self-reported weight and height. For adults, the World Health Organization (WHO) defines overweight and obesity as follows: overweight is a BMI ≥ 25 kg/m^2^, and obesity is a BMI ≥ 30 kg/m^2^ [17].

Blood samples were collected from 308 soldiers and civilian military personnel in Polish military units between October 2023 and February 2024. The collected blood samples were stored at 2–8 °C and sent to the laboratory for lipid profile testing.

The aim of this study was to assess the occurrence of risk factors for cardiovascular disease episodes and to assess the self-awareness of study participants regarding the risk factors for cardiovascular disease.

### Statistical Analysis

Excel tools (Version 2507 Build 16.0.19029.20136) and Statistica 13.3 software were used for statistical processing of the collected material. Descriptive statistics and parametric and nonparametric data analysis methods were used. The Shapiro–Wilk test was used to assess the normality of the distribution. The Mann–Whitney U test was used to assess the relationship between quantitative variables in the absence of normal distribution. The Kruskal–Wallis H test and post hoc analysis: multiple comparison of mean ranks for all samples were used to analyze more than two groups of an ordinal variable. The assessment of the relationship between two qualitative variables was performed using the Pearson Chi^2^ test of independence. The correlation coefficient was calculated to express the interdependence between two variables. The r-Pearson correlation coefficient was used to describe the correlation between quantitative variables, and the Spearman rank correlation coefficient was used in the absence of normal distribution. The level of significance was considered to be *p* ≤ 0.05.

The analyses were conducted by gender, age (<20; ≥30 and <40; ≥40 and <50; ≥50 and <60; ≥60 years), professional group, and military service corps.

Only statistically significant results are presented in the figures. The tables show the results of analyses by the military service corps. Due to the extensive amount of data obtained, the remaining sub-analyses are described in the text.

## 3. Results

### Characteristics of the Respondents

The study group consisted of 308 people, including 253 soldiers (82.14%) and 55 civilian employees of the army (17.86%). More than 2/3 were men (69.16%). The respondents were aged 20–67 ($\bar{x}$ = 42.83 ± 5.29; Q1 = 35.00 and Q3 = 49.00); 61.04% of people had higher education, 36.04% secondary, and the rest vocational or primary. Below is a detailed description of quantitative variables constituting risk factors for cardiovascular diseases (Table 1).

**Figure 1:**
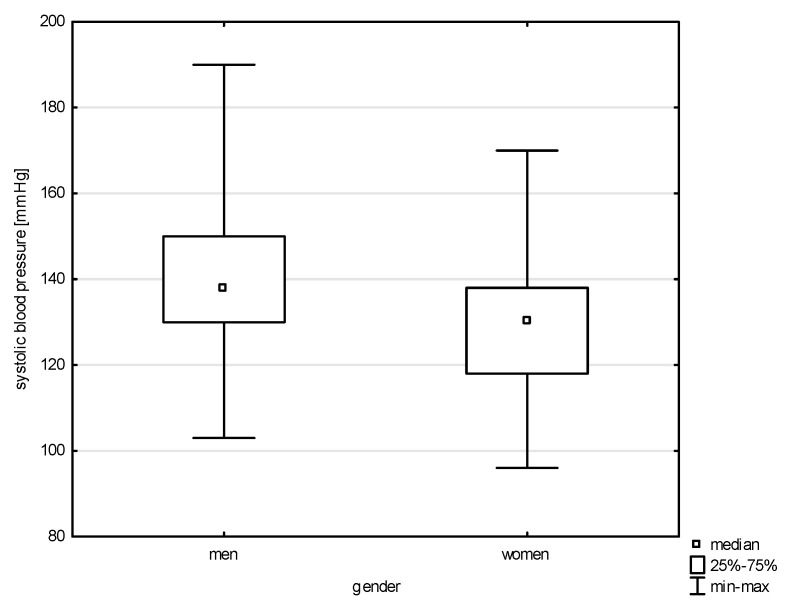
Relationship between systolic BP and gender (*p* < 0.0000).

**Figure 2:**
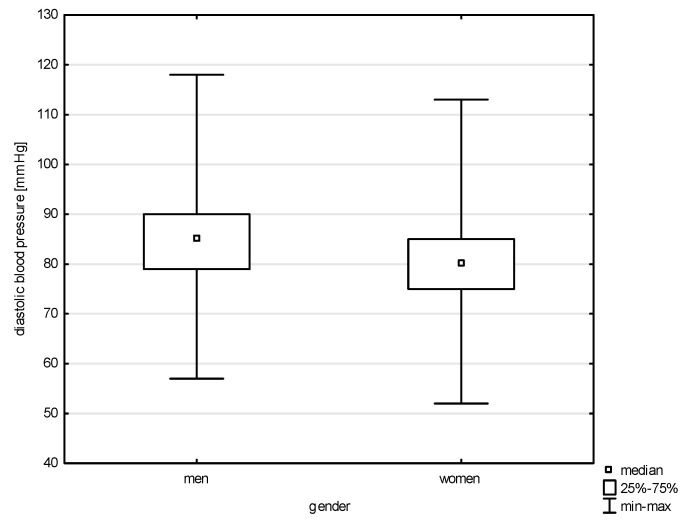
Relationship between diastolic BP and gender (*p* < 0.0000).

**Figure 3:**
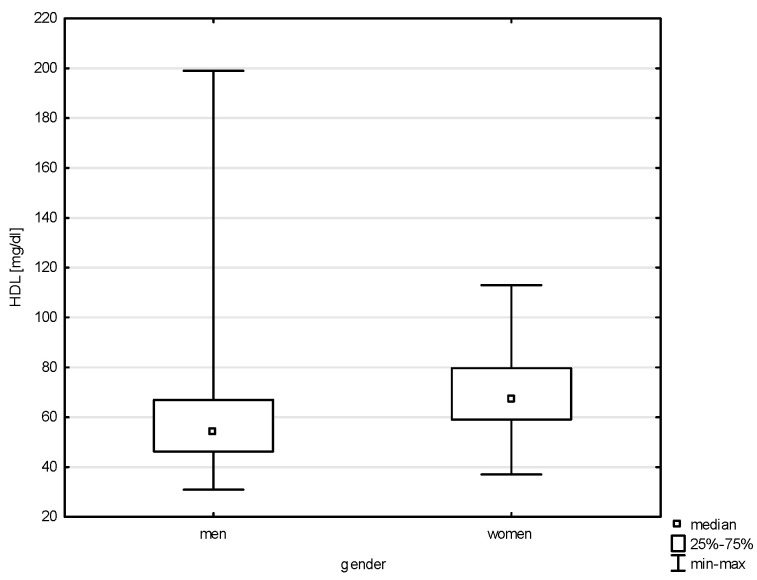
Relationship between HDL and gender (*p* < 0.0000).

**Figure 4:**
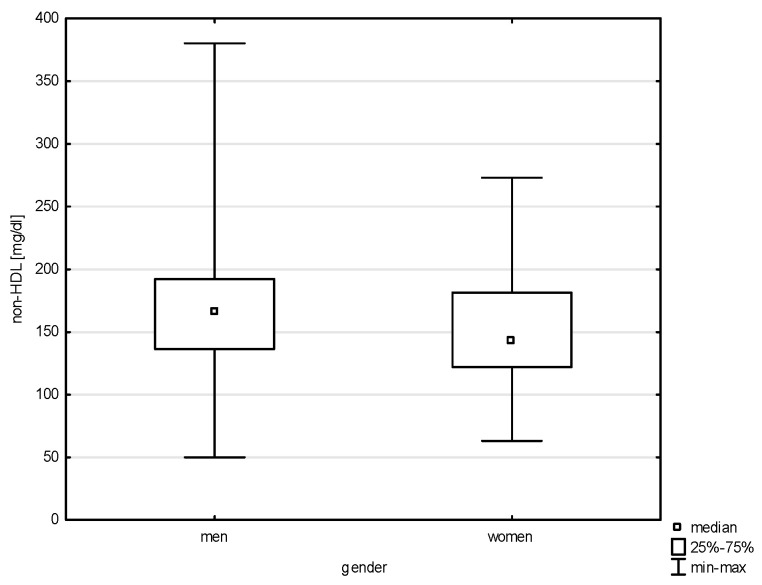
Relationship between non-HDL and gender (*p* = 0.0031).

**Figure 5:**
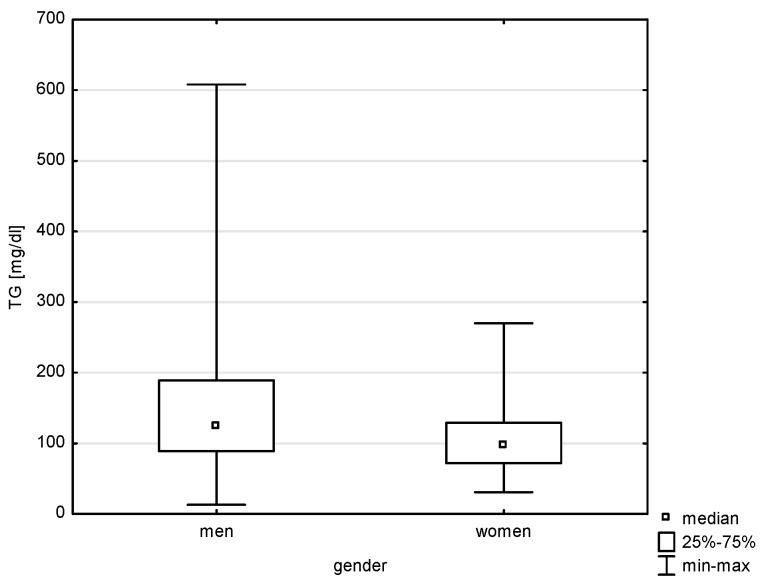
Relationship between TG and gender (*p* < 0.0000).

This study analyzed the socio-demographic variables of the respondents in correlation with quantitative risk factors of cardiovascular diseases. Significant differences were found between the variable gender and all of the above-mentioned factors except LDL (Z = 1.543, *p* = 0.1225) and total cholesterol (Z = 0.768, *p* = 0.4423). Statistical significance was noted with a weak correlation between gender and systolic blood pressure (BP) (Z = 5.100, *p* = 0.0000, rho = 0.291), diastolic blood pressure (Z = 4.151, *p* = 0.0000, rho = 0.238), HDL (Z = 6.030, *p* = 0.0000, rho = 0.344), non-HDL (Z = 2.950, *p* = 0.0031, rho = 0.168), and TG (Z = 4.204, *p* = 0.0000, rho = 0.240), as shown in Figure 1, Figure 2, Figure 3, Figure 4 and Figure 5. Moreover, 61.17% of men and 68.97% of women admitted to smoking in the past, which did not constitute a significant correlation (Chi^2^ = 1.605, *p* = 0.205). Currently, the phenomenon of smoking tobacco occurs more often among men than women, i.e., 23.44% and 15.91% (Chi^2^ = 2.104, df = 1, *p* = 0.1468).

**Figure 6:**
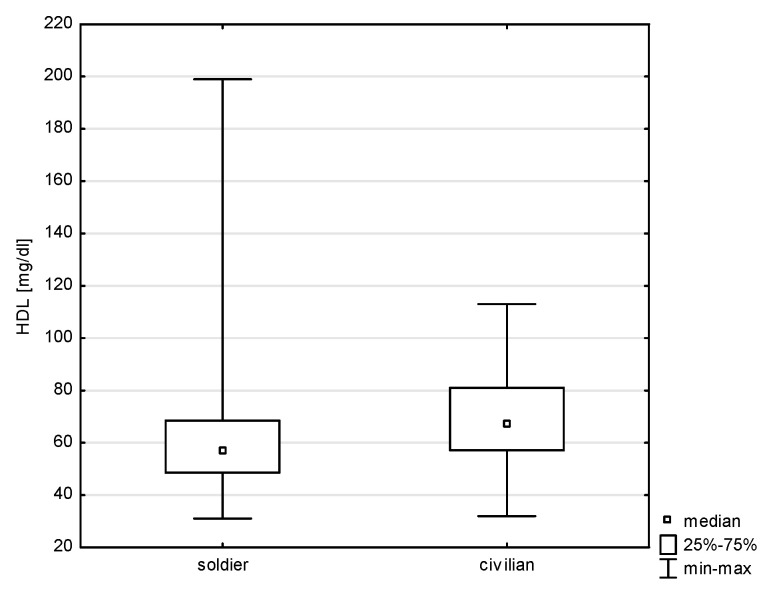
Relationship between HDL and professional group (*p* < 0.0000).

The applied analysis did not show any correlation between the study groups (soldier/civilian employee) and almost all cardiovascular risk factors, except for the HDL parameter (Z = 3.940, *p* = 0.0000; rho = 0.225, *p* = 0.0000), as shown in Figure 6. Almost the same percentage of past smokers were concerned soldiers and civilian employees, i.e., 63.37% and 64.00%, respectively. Current smoking was declared by 22.27% of soldiers and 16.00% of civilians (Chi^2^ = 0.977, df = 1, *p* = 0.3228).

**Figure 7:**
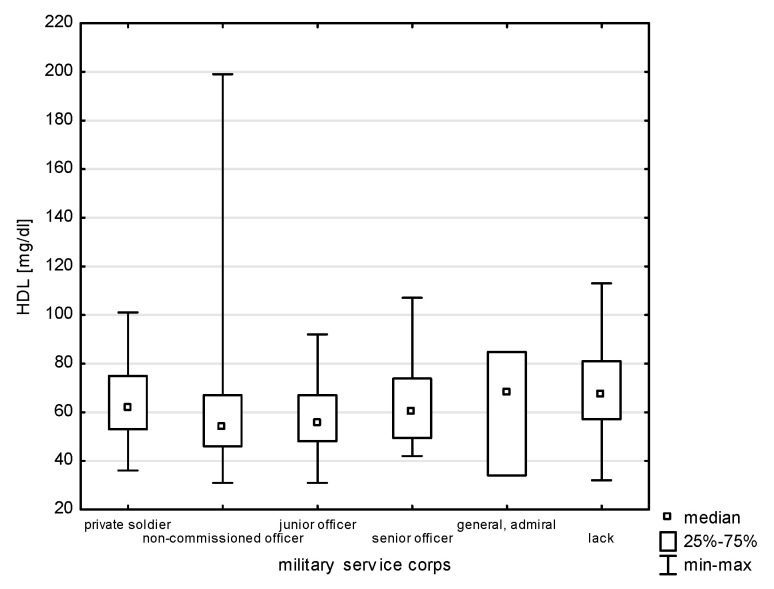
Relationship between HDL and military service corps (*p* = 0.0002).

**Figure 8:**
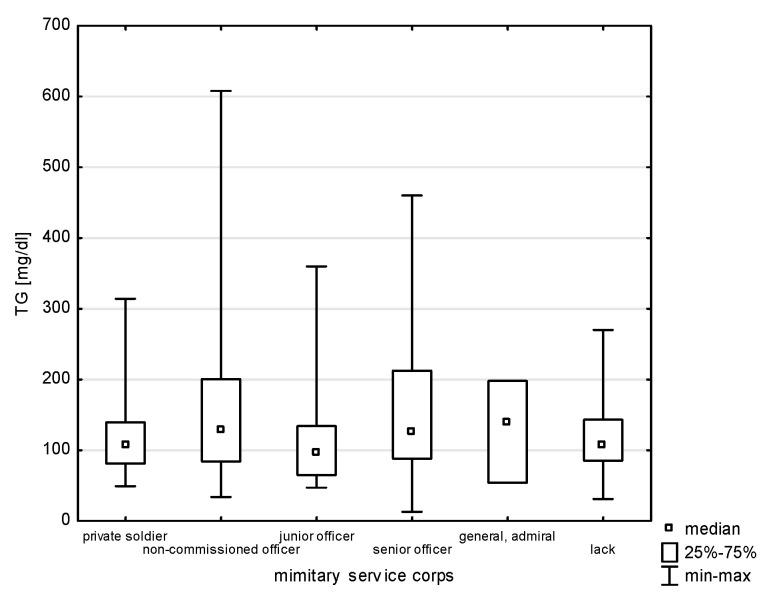
Relationship between non-TG and military service corps (*p* = 0.0070).

A relationship was found between the professional corps and selected risk factors of heart diseases, i.e., HDL (H = 24.323, *p* = 0.0002) and TG (H = 15.922, *p* = 0.0070), without significant differences for multiple comparisons (Figure 7 and Figure 8). The largest group of past smokers comprised non-commissioned officers (35.48%); every fourth was a junior officer (24.73%), and 13.98% a senior officer (Chi^2^ = 14.040, *p* = 0.0153). Current smoking was also most frequently declared by non-commissioned officers (44.44%) and a similar percentage of senior officers and junior officers, i.e., 19.05% and 14.29% (Chi^2^ = 5.502, df = 5, *p* = 0.3576).

**Figure 9:**
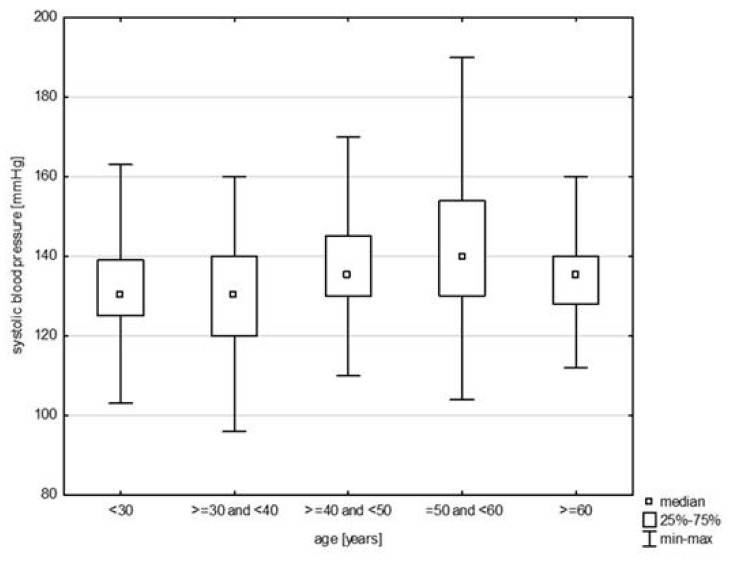
Relationship between systolic BP and age (*p* = 0.0008).

**Figure 10:**
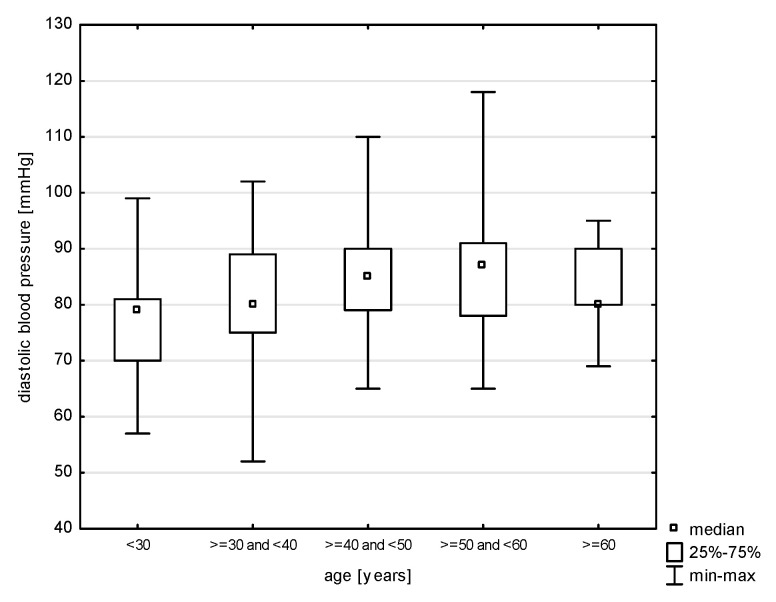
Relationship between diastolic BP and age (*p* = 0.0018).

**Figure 11:**
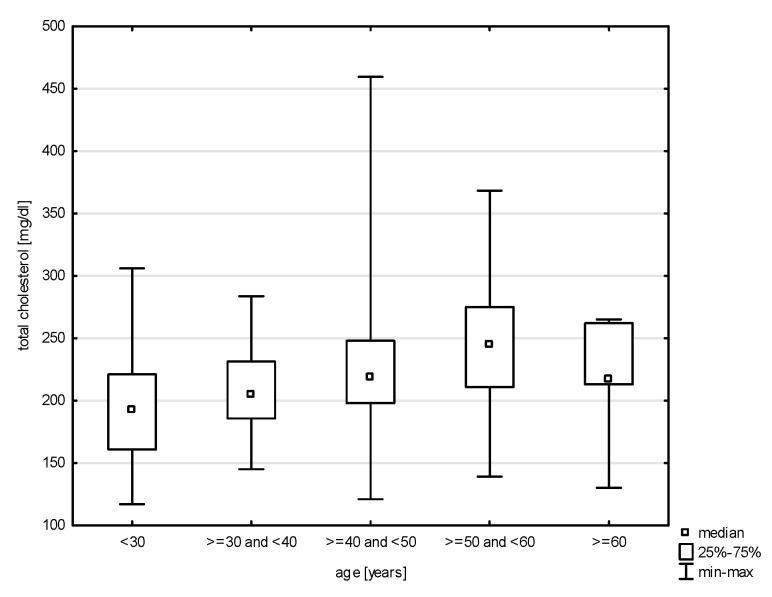
Relationship between total cholesterol and age (*p* < 0.0000).

**Figure 12:**
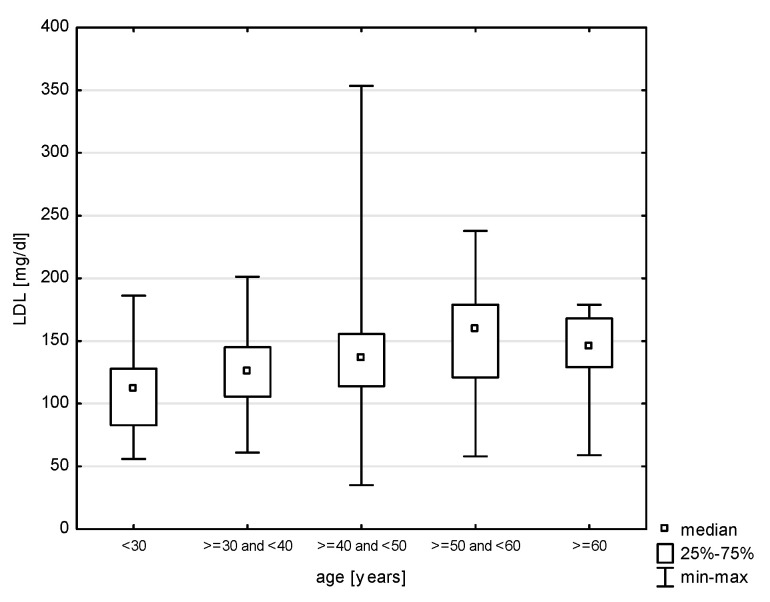
Relationship between LDL and age (*p* < 0.0000).

**Figure 13:**
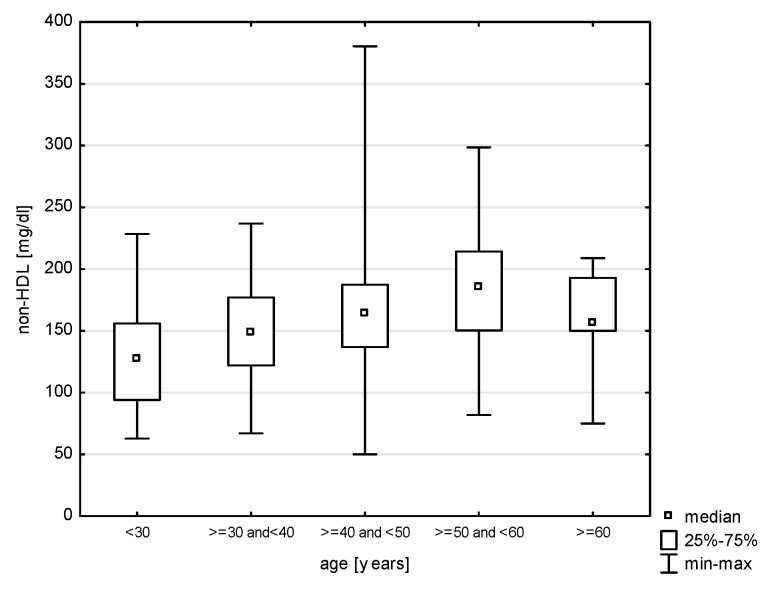
Relationship between non-HDL and age (*p* < 0.0000).

**Figure 14:**
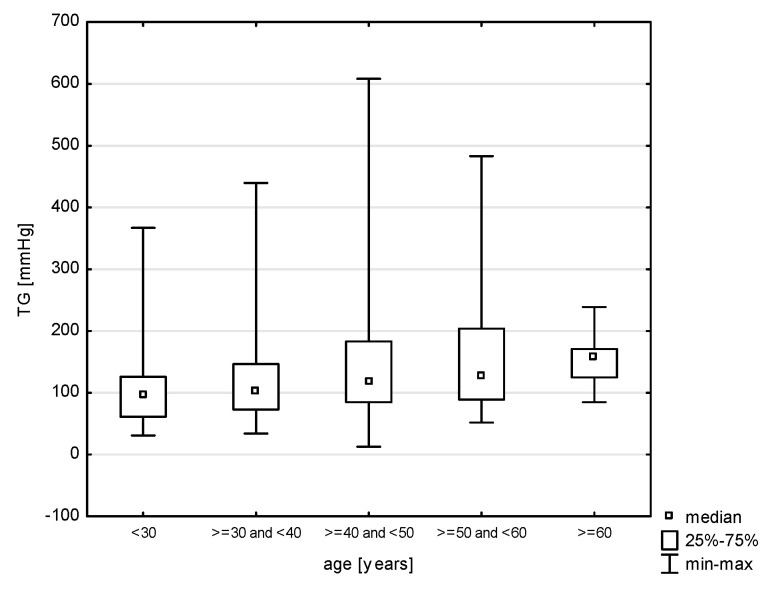
Relationship between TG and age (*p* < 0.0046).

In the division of the study group into age groups, a relationship with systolic blood pressure was demonstrated (H = 19.079, *p* = 0.0008). The post hoc test showed significant rank differences between the age group of 30–40 years and the persons in the age group of 40–50 (*p* = 0.0264) years and 50–60 years (*p* = 0.0006) (Figure 9). In turn, in the relationship with diastolic blood pressure (H = 17.217, *p* = 0.0018), differences were observed between the youngest study subjects and the age group of 30–40 years (*p* = 0.0015) and 40–50 years (*p* = 0.0000) and between 30–40 years and 50–60 years (*p* = 0.0002) (Figure 10). Also, the relationship with the total cholesterol variable turned out to be statistically significant (Z = 37.965, *p* = 0.0000), and the post hoc test showed differences between the youngest age group and people aged 40–50 (*p* = 0.0048) and 50–60 (*p* = 0.0000), as well as between 30–40 and 50–60 (*p* = 0.0000) and 40–50 and 50–60 (*p* = 0.0398) (Figure 11). In the correlation with the LDL variable (H = 34.574, *p* = 0.0000), significant rank differences were noted among people under 30 and 40–50 year olds (*p* = 0.0048) and 50–60 year olds (*p* = 0.0000), as well as among 30–40 year olds and 50–60 year olds (Figure 12). Also, in the non-HDL variable (Z = 34.618, *p* = 0.0000), the post hoc test showed dependencies in the same groups, for which the *p*-value was, respectively, 0.0010, 0.0000, and 0.0003 (Figure 13). In turn, in the analysis with the TG variable (Z = 15.068, *p* = 0.0046), significance was observed only between the youngest and 30–40 year-olds (*p* = 0.0324) and 50–60 year-olds (*p* = 0.0108) (Figure 14). The largest group of past smokers was people aged 40–50 (Chi^2^ = 10.848, df = 4, *p* = 0.0283). Currently, there is almost the same percentage of people aged 40–50 and 50–60 who smoke, i.e., 36.51% and 33.33% (Chi^2^ = 9.796, df = 4, *p* = 0.0440).

A positive family history of circulatory system diseases was declared by 56.04% of women and 38.57% of men (Chi^2^ = 8.720, df = 2, *p* = 0.0127), and 40.73% of soldiers and 58.49% of civilian employees of the army (Chi^2^ = 5.709, df = 2, *p* = 0.0575). Four out of ten respondents who had a family member with cardiovascular disease were aged 40–50, and every fifth was aged 30–40 (Chi^2^ = 8.3327, df = 8, *p* = 0.4021). There was no significance in the division into professional corps of the respondents (Chi^2^ = 14.579, df = 10, *p* = 0.1481); however, they were most frequently reported by non-commissioned officers (40.91%) and almost the same number of senior and junior officers, i.e., 15.91% and 13.64%, respectively.

A total of 22.85% (n = 69) of all respondents, including 20.78% of women and 23.70% of men, suffer from circulatory system diseases or have no knowledge in this area (Chi^2^ = 0.837, df = 2, *p* = 0.6580). This phenomenon occurs in a comparable percentage among soldiers and civilian employees, i.e., 22.09% and 26.41% (Chi^2^ = 0.514, df = 2, *p* = 0.7733). The highest percentage of those diagnosed with cardiovascular disease or having no opinion on this subject was among senior officers (47.37%), followed by non-commissioned officers at 23.62% (Chi^2^ = 24.008, df = 10, *p* = 0.0075).

The frequency of selected symptoms of cardiovascular diseases was analyzed. Most respondents declared the absence of the above symptoms. Chest pain was reported by 8.94% of the respondents, including 14.29% of women and 6.64% of men (Chi^2^ = 4.571, df = 1, *p* = 0.0325), and 8.84% of soldiers and 9.43% of civilians (Chi^2^ = 0.0192, df = 1, *p* = 0.8897). The feeling of shortness of breath was declared by 4.97% of the population, being almost four times less often in soldiers than civilian employees of the army, i.e., 3.61% and 11.32% (Chi^2^ = 5.497, df = 1, *p* = 0.0190) and almost 10 times more often in women than men, i.e., 13.19% and 1.42% (Chi^2^ = 18.644, df = 1, *p* = 0.0000). Dizziness was reported by 6.95% of respondents, being 5 times more often in women than in men, i.e., 15.38% and 3.32% (Chi^2^ = 14.309, df = 1, *p* = 0.0001) and 2.5 times less often in soldiers than in civilians, i.e., 5.62% and 13.21% (Chi^2^ = 3.885, df = 1, *p* = 0.0487). Heart palpitations were reported by 11.26% of the population, including 18.68% of women and 8.06% of men (Chi^2^ = 7.183, df = 1, *p* = 0.0073). Almost half as many soldiers (10.04%) and civilian employees (16.98%) declared the occurrence of the above-mentioned symptom (Chi^2^ = 2.107, df = 1, *p* = 0.1466). Pain in the lower limbs was declared by 7.95% of the respondents, with three times more often in women than men, i.e., 14.29% and 5.21% (Chi^2^ = 7.153, df = 1, *p* = 0.0074). This symptom was almost twice as often reported by civilians as by soldiers, i.e., 15.09% and 6.43% (Chi^2^ = 4.488, df = 1, *p* = 0.0341). Almost 1/3 of the population (29.47%) experienced headache, including 41.76% of women and 24.17% of men (Chi^2^ = 9.461, df = 1, *p* = 0.0021). In a significant percentage, civilians reported this symptom more often than soldiers, i.e., 41.51% and 26.91% (Chi^2^ = 4.482, df = 1, *p* = 0.0342). Increased blood pressure was declared by almost every fifth (19.21%) of the examined persons, happenning more often in men than in women, i.e., 22.27% and 12.09% (Chi^2^ = 4.252, df = 1, *p* = 0.0392) and more often in soldiers than in civilians, i.e., 20.48% and 13.21% (Chi^2^ = 1.490, df = 1, *p* = 0.2221). In the analysis, divided into military service corps depending on the occurrence of symptoms of cardiovascular diseases (Table 2), no statistical significance was noted.

The frequency of selected risk factors of cardiovascular diseases was analyzed in the study participants. They were present with moderate frequency in most respondents. Overweight was reported by 30.30% of the study participants, including 19.32% of women and 34.93% of men (Chi^2^ = 7.144, df = 1, *p* = 0.0075), and 31.98% of soldiers and 22.00% of civilians (Chi^2^ = 1.962, df = 1, *p* = 0.1612). Statistical significance was demonstrated between the analyzed variable and age groups (Z = 2.147, *p* = 0.0238) with a very weak correlation (rho = 0.131). In turn, obesity was declared by almost every seventh (14.81%) of the surveyed, including 17.05% of women and 13.88% of men (Chi^2^ = 0.482, df = 1, *p* = 0.4873) and 14.17% of uniformed services and 18.00% of civilians (Chi^2^ = 0.462, df = 1, *p* = 0.44962), without significance between age groups (Z = 1.771, *p* = 0.0764). No differences were observed in the consumption of an incorrect diet (38.72%), which was reported by 34.09% of women and 40.67% of men (Chi^2^ = 1.129, df = 1, *p* = 0.2878), and almost the same was reported for soldiers and civilian employees, i.e., 38.87% and 38.00%. However, significance was demonstrated in the division into age groups (Z = 2.257, *p* = 0.0175) with a very weak correlation (rho = 0.138). Increased cholesterol level occurred in almost every sixth examined person (17.17%), almost with the same frequency among women (15.91%) and men (17.70%), as well as soldiers and civilians, i.e., 16.19% and 22.00% (Chi^2^ = 0.985, df = 1, *p* = 0.3206). In turn, significance was demonstrated in the age groups (Z = 2.908, *p* = 0.0022) with an almost weak correlation (rho = 0.177). Low physical activity was declared by every fifth respondent (21.21%), with almost twice as often by women than men, i.e., 31.82% and 16.75% (Chi^2^ = 8.417, df = 1, *p* = 0.0037). Every sixth soldier and over every third civilian also indicated the occurrence of this factor, i.e., 17.81% and 38.00% (Chi^2^ = 10.138, df = 1, *p* = 0.0014). In the classification of the respondents according to age groups, no correlation was found (Z = 0.659, *p* = 0.4876). Almost 1/5 of the studied population (21.21%) declared smoking tobacco, with a slight difference between women and men, i.e., 15.91% and 23.44% (Chi^2^ = 2.104, df = 1, *p* = 0.146). Soldiers indicated smoking tobacco slightly more often than civilian employees, i.e., 22.27% and 16.00% (Chi^2^ = 0.977, df = 1, *p* = 0.3228). However, the analysis by age groups turned out to be statistically significant (Z = 2.013, *p* = 0.0440) with a very weak correlation (rho = 0.123). A total of 15.49% of the respondents admitted to drinking alcohol, which was three times more often in men than women (19.14% vs. 6.82%) and four times more often in soldiers than civilians (17.81% vs. 4.00%). In both of the above analyses, the result was statistically significant, i.e., Chi^2^ = 7.181, df = 1, *p* = 0.0073 and Chi^2^ = 6.062, df = 1, *p* = 0.0138. In turn, in age groups, significance was observed (Z = 2.413, *p* = 0.0108) without significant correlation (rho = 0.147). More than half (54.21%) of respondents indicated the occurrence of stress exposure, without differences in the division into professional group, i.e., soldiers 54.25% and civilians 54.00% and without significance by age group (Z = 1.063, *p* = 0.2873). In turn, in the division by gender, a significantly higher percentage of women (62.50%) than men (50.72%) indicated this risk factor (Chi^2^ = 3.463, df = 1, *p* = 0.0627). In most analyses, in the division into military service corps depending on the occurrence of risk factors (Table 3), no statistical significance was noted. However, a relationship was observed between the occurrence of overweight (Chi^2^ = 12.260, df = 5, *p* = 0.0313) and low physical activity (Chi^2^ = 12.847, df = 5, *p* = 0.0248) and the military service corps.

Respondents assessed their health condition on a five-point scale (5—very good; 4—good; 3—sufficient; 2—poor; 1—no opinion). More than half (54.88%) of respondents assessed their health as good, 27.61% as very good, 9.76% as satisfactory, and 0.34% as poor. Significant differences were noted by gender (Chi^2^ = 10.278, df = 4, *p* = 0.0359) and professional group (Chi^2^ = 10.171, df = 4, *p* = 0.0376). A comparable percentage of men and women assessed their health as very good, i.e., 28.23% vs. 26.14%. A significantly higher percentage of women (14.77%) than men (4.31%) had no opinion on their health. In turn, in the division by professional group, soldiers more often than civilian employees assessed their health as good, i.e., 30.36% vs. 14.00%. Twice as many civilians (16.00%) as soldiers (8.50%) had a satisfactory opinion about their health condition. The analysis by age group also proved statistically significant (H = 23.602, *p* = 0.0001), and the post hoc test showed a correlation between the youngest group and people aged 30–40 (*p* = 0.0007), 40–50 (0.0132), and over 60 (*p* = 0.0170). In addition, a relationship was noted between the assessment of health and the military service corps (Table 4), the test value of which was Chi^2^ = 54.706, df = 20, *p* = 0.0000.

The study participants were asked to assess their knowledge of circulatory system diseases using a five-point scale (5—very good; 4—good; 3—sufficient; 2—below sufficient; 1—no opinion). More than half of the respondents declared sufficient (56.27%) knowledge on the subject, and every fourth respondent—good. There was no significant difference in terms of gender (Chi^2^ = 4.738, df = 4, *p* = 0.3151). A comparable percentage of men and women described their knowledge as sufficient, i.e., 53.41% vs. 57.49%. A slightly higher percentage of men (26.57%) than women (21.59%) declared a good level of knowledge. Significance was demonstrated in the division into professional groups (Chi^2^ = 11.121, df = 4, *p* = 0.0252). Every fourth soldier (24.08%) and almost every third civilian (30.00%) assessed their knowledge as good. In turn, more than half of the military (59.18%) and 42.00% of the remaining respondents have sufficient information in this area. The result in the division into age groups turned out to be insignificant (H = 2.673, *p* = 0.6139), as well as in the military service corps (Chi^2^ = 20.778, df = 20, *p* = 0.4102), Table 5.

The majority of respondents (67.79%) expressed a desire to expand their knowledge about cardiovascular diseases and their risk factors, while every fourth person had no opinion (24.50%). No significant differences were noted with regard to gender (Chi^2^ = 2.992, df = 2, *p* = 0.2239) and professional group (Chi^2^ = 1.686, df = 2, *p* = 0.4302). Men were twice as likely as women (9.05% vs. 4.55%) not to show willingness to expand their knowledge in this area, with a slightly higher percentage of soldiers than civilians (8.10% vs. 5.88%). In addition, every fourth person serving in the military and almost every third civilian employee had no opinion on the subject. The result in the division into age groups turned out to be insignificant (H = 2.824, *p* = 0.2436), as well as in the military service corps (Chi^2^ = 8.734, df = 10, *p* = 0.5574), Table 6.

## 4. Discussion

A total of 22.85% of all respondents in our study—including 20.78% of women and 23.70% of men—reported having cardiovascular diseases or lacking knowledge about their condition. Most participants did not report cardiovascular symptoms; however, those who did were more often civilian employees and women. Shortness of breath was nearly four times less common among soldiers than civilians and nearly ten times more common among women than men. Dizziness was reported five times more often by women than by men and 2.5 times less frequently by soldiers than civilians. Only elevated blood pressure was reported more frequently in men than women and more often in soldiers than civilians. Moreover, in our study, statistically significant differences by gender were observed for systolic and diastolic blood pressure, non-HDL cholesterol, and triglyceride levels. In addition to these differences in symptom prevalence, our analysis revealed important associations between age, gender, and cardiovascular risk factors.

Our study also confirms a statistically significant relationship between age group and systolic blood pressure. Post hoc testing revealed significant rank differences between the 30–40 age group and those aged 40–50 and 50–60. Similar age-related trends were observed for diastolic blood pressure. Gender differences were also evident. In the studied population, nearly 69.00% of women and slightly more than 61% of men reported having smoked in the past. At the time of the survey, 21.10% of respondents reported current smoking, with a higher prevalence among soldiers (22.27%) than among civilians (16.00%).

Our findings confirm a significant association between age and lipid parameters, with the most pronounced differences observed in total cholesterol, LDL-C, and non-HDL levels. These were significantly higher among older individuals compared to younger age groups, indicating an age-related increase in lipid abnormalities, consistent with the existing literature on metabolic changes across the lifespan. Among modifiable cardiovascular risk factors, most—such as physical inactivity, alcohol consumption, tobacco use, elevated blood pressure, and diabetes—are more frequent in men than in women. While most analyses in our study did not find significant associations between military service corps and cardiovascular risk factors, a relationship was observed between service corps and both overweight people and people with low physical activity. In our study, elevated cholesterol was found in nearly one in six participants (17.17%), with similar frequencies across gender and between military and civilian respondents.

Obesity is the only risk factor more prevalent in women [4], which aligns with our observations: 17.05% of women and 13.88% of men were classified as obese (with no significant differences across age groups). Overweight was reported by 30.30% of participants—19.32% of women and 34.93% of men—as well as by 31.98% of soldiers and 22.00% of civilians. A statistically significant but weak association between overweight and age group was observed. There was also overrepresentation and underrepresentation of certain military ranks in CVD diagnoses and associated risk factors, indicating an unequal distribution of risk within the study population. Non-commissioned officers and senior officers had disproportionately high rates of hypertension and hypercholesterolemia relative to their representation in the sample—possibly due to higher age, greater occupational stress, or better access to medical care. Conversely, privates were underrepresented, which may reflect their younger age, higher physical activity levels, or underdiagnosis [18].

Our findings indicate that approximately 10% of respondents rated their health status as fair or poor. Notably, soldiers were more likely than civilian employees to rate their health as good (30.36% vs. 14.00%), while twice as many civilians (16.00%) as soldiers (8.50%) rated their health as fair. A statistically significant difference was also observed across age groups. Moreover, over half of the participants assessed their knowledge of CVD as sufficient (56.27%), and one in four rated it as good. No gender differences were observed in self-assessed knowledge, but significant differences emerged between occupational groups—civilians tended to rate their knowledge higher than soldiers. Encouragingly, the vast majority of respondents (67.79%) expressed a willingness to expand their knowledge about cardiovascular diseases and their risk factors (with no significant differences by gender or occupation). These findings underscore the ongoing need for targeted educational initiatives in cardiovascular prevention and early self-assessment. This need is further supported by the observed gaps in specific risk factor awareness. Similar to the findings of a study conducted in Southern Iran based on the Protection Motivation Theory (PMT), effective CVD prevention strategies should combine health education with actions that strengthen self-efficacy and protection motivation, particularly regarding diet and regular physical activity, which can significantly increase the willingness to engage in health-promoting behaviors [19].

Taken together, these patterns indicate the need for a differentiated approach to CVD prevention and diagnosis within the armed forces, tailored to both military rank and the specific demands of service. When placing these findings in the context of global and military health trends, several important patterns emerge. Tobacco smoking is one of the most significant modifiable risk factors for cardiovascular diseases (CVD). Central and Eastern Europe currently bears the highest global burden of CVD attributable to smoking. It is estimated that smoking causes approximately 6 million deaths worldwide each year. In this region, the age-standardized mortality rate reaches 77.82 per 100,000 population, significantly exceeding values observed in other parts of the world. In 2019, the absolute number of CVD deaths attributable to smoking was four times higher among men than women [3]. The most substantial age-standardized decline in tobacco use is expected in the Americas (an average relative decrease of 33.00% between 2010 and 2025), while the slowest decline is anticipated in the Western Pacific Region (an average relative decrease of 8% in the same period) [5]. Nieh et al. demonstrated that members of the United States Armed Forces smoke more frequently than the general population, with even higher smoking prevalence observed among American veterans [20]. Similarly, Al-Khashan emphasized the high frequency of smoking among military personnel in Saudi Arabia, particularly within the Navy and air force, as well as among younger enlisted ranks, individuals with lower education levels, lower income, and those who were divorced or widowed [11]. A downward trend in smoking prevalence has also been observed in the South Korean military, where the percentage of professional soldiers who smoke decreased from 46.20% in 2008 to 34.10% in 2022. In the study by Jung et al., the highest smoking rates were reported among individuals born in the 1970s, while those born after 1980 showed significantly lower rates. The authors emphasized that military service itself contributes substantially to the increase in smoking; men who served in the military were more likely to smoke than their civilian peers. This suggests a critical role of occupational stressors such as work-related stress, separation from family, or the pressure to conform to military culture [21].

The coexistence of smoking and obesity, frequently observed in individuals with hypertension, contributes to difficult-to-control blood pressure. Mechanistically, this includes increased sympathetic nervous system activation, endothelial dysfunction, chronic inflammation, oxidative stress, and insulin resistance. Therefore, a multidimensional approach involving lifestyle modification, antihypertensive pharmacotherapy, and smoking cessation interventions is recommended [22].

Although premature cardiovascular mortality has decreased in both women and men since 2000, cardiovascular diseases remain the leading cause of death in Poland, affecting men and women at different life stages—beginning after age 44 in men. Men are burdened with 2.5 times higher premature mortality compared to women [20]. These findings are consistent with data from the Institute for Health Metrics and Evaluation (IHME), which reported 1200% more CVD-related deaths in men than in women in 2021 [5]. These observations are consistent with World Heart Federation statistics, which highlight sex-based disparities in cardiovascular risk. Elevated blood pressure occurs in 34.60% of men compared to 23.00% of women, representing a 50% higher prevalence in males [4]. High systolic blood pressure is the most important global risk factor for CVD and was associated with approximately 10.8 million deaths in 2021. It also contributes most significantly to years of life lost due to cardiovascular disease, amounting to 2564.9 DALYs per 100,000 population [4,5]. This gender disparity in the prevalence of hypertension contributes to the overall greater cardiovascular burden among men and aligns with broader European trends, where high blood pressure is the leading risk factor for about 24.00% of cardiovascular deaths [5]. Among U.S. veterans, 71.00% had diagnosed hypertension, and 66% had uncontrolled blood pressure based on Veterans Health Administration (VHA) guidelines. The majority of veterans with uncontrolled blood pressure and at least one additional cardiovascular risk factor remained hypertensive even after five years of follow-up [23]. Research by Gielerak revealed a clear increase in the prevalence of hypertension with age, diagnosed in 44.70% of Polish soldiers over the age of 50. More than 50.00% of cases showed elevated blood pressure values in office measurements, and 14.00% of cases qualified as stages 2 or 3 hypertension. Among soldiers with normal blood pressure, one-third had borderline values. Additionally, 14.00% reported a history of hypertension, of whom 86% exhibited elevated values during the examination [7]. Hypertension affects 14.70% of Hungarian military pilots [24] and 36.30% of military pilots in Serbia [25]. Between 2007 and 2016, the prevalence of diagnosed hypertension in the overall U.S. military population was 15.3 per 100 personnel—14.6 in the Air Force and 9.7 among pilots and crew members [26].

A key driver of atherosclerotic cardiovascular disease (ASCVD) is dysregulation in low-density lipoprotein (LDL) metabolism. The concentration of LDL cholesterol (LDL-C) remains a primary diagnostic marker for identifying lipid disorders (hypercholesterolemia) and for monitoring lipid-lowering therapy. Non-HDL cholesterol and apolipoprotein B (apoB) levels serve similar diagnostic roles, though non-HDL cholesterol—which reflects the total amount of atherogenic lipoproteins in the blood—is considered a better predictor of cardiovascular risk than LDL-C alone [27]. Lipid disorders remain a major diagnostic and therapeutic challenge [27]. Studies on atherosclerosis indicate that long-term exposure to elevated LDL cholesterol levels is a key risk factor for cardiovascular disease [28]. In Poland, depending on sampling methodology, the prevalence of dyslipidemia in adults is estimated at 60–80%, with nearly 20 million individuals suffering from hypercholesterolemia. Most remain unaware of their condition, and only about 5% of the estimated 140,000 patients with familial hypercholesterolemia (FH) have been diagnosed [27,28]. While hypercholesterolemia has declined in Western Europe, this trend does not apply to Central and Eastern Europe, where the prevalence of type 2 diabetes and obesity among men continues to rise [1]. In a large-scale study of Polish soldiers, more than half (52%) had elevated total cholesterol, and 60% had elevated LDL-C levels—the main contributor to atherosclerosis and CVD [7].

In cases of lipid disorders, current recommendations emphasize intensive lipid-lowering therapy—not only with statins, but also with newer treatment options—under the principles of “the lower the better”, “the earlier the better”, and “the longer the better.” Such approaches can reduce cardiovascular event risk by as much as 50–55% in patients [24]. Individuals with untreated hypercholesterolemia are at significantly higher risk for cardiovascular events, which are often asymptomatic until a first incident such as myocardial infarction, stroke, or peripheral artery disease occurs [29]. National Polish studies such as NATPOL III PLUS and WOBASZ report similar findings: hypercholesterolemia affected 59.50% of men and 62.00% of women in NATPOL, and 67.00% of men and 64.00% of women in WOBASZ [27]. Our results highlight the importance of early lipid profile monitoring, particularly among individuals entering their fourth and fifth decades of life, when the risk of lipid disorders increases significantly.

A 2023 Iranian study confirmed that diabetes, hyperlipidemia, hypertension, obesity, and metabolic syndrome are major cardiovascular risk factors among soldiers. Although the prevalence of metabolic syndrome in this group was lower than in peers from other countries, other CVD risk factors were common. Notably, the rate of obesity among military personnel was similar to that in the general population [30].

Lifestyle modification is recommended for all patients, including those on pharmacological therapy. Lipid-lowering drugs are advised only in selected clinical scenarios [27]. Movsisyan et al. estimated that more than half of the decline in ischemic heart disease mortality can be attributed to reductions in major risk factors, with less than half attributable to evidence-based treatment [31]. The 2023 study highlights the prevalence of CVD diagnoses in military populations and reveals that only 23.40% of respondents (N = 311) demonstrated knowledge of most or all major cardiovascular risk factors—such as elevated cholesterol levels, tobacco smoking, advanced age, abdominal obesity, and alcohol abuse. Still, respondents showed a generally sufficient understanding of the factors influencing blood cholesterol levels, providing a solid foundation for developing more comprehensive educational interventions [32]. Globally, the most frequently implemented tool for combating tobacco use is the national tobacco control program, currently in place in 91% of countries. These are followed by clinical guidelines, protocols, and standards for cardiovascular disease management (86.00%) and national policies, strategies, or action plans targeting unhealthy diets linked to non-communicable diseases (85.00%). The least implemented intervention is the plan to reduce harmful alcohol consumption, existing in only 70.00% of countries [4]. Special attention should be paid to the rising popularity of electronic cigarettes. While the prevalence of traditional cigarette smoking continues to decline, the use of e-cigarettes is increasing among adults. According to Espinoza-Derout et al., in 2020, 19.60% of high school students in the United States reported using e-cigarettes. Regular use was associated with elevated biomarkers indicating sympathetic nervous system activation, oxidative stress, inflammation, endothelial dysfunction, and potential prothrombotic effects, although prospective data are lacking [33]. This phenomenon is particularly concerning given the increasing popularity of these products among school-age youth, who represent the future pool of military service candidates.

These findings provide important guidance for developing effective cardiovascular prevention programs tailored to the unique characteristics of military and civilian personnel. While certain protective patterns are visible—such as relatively high health self-assessment and willingness to expand knowledge—the persistence of modifiable risk factors like smoking, dyslipidemia, and obesity underscores the urgent need for targeted interventions. Integrating regular health monitoring with tailored education programs by gender, age, and military service corps may be key to reducing the cardiovascular burden in this population.

This study highlights significant differences in cardiovascular risk profiles within military and civilian personnel, emphasizing the urgent need for tailored preventive strategies and targeted education to address modifiable risk factors in this population.

The presented results can be considered a pilot study, indicating the need for further research on a representative population.

This study has several limitations that should be considered when interpreting the results:

1. This study was based on a relatively small sample size, which may limit the statistical power of the analyses and reduce the generalizability of the findings to a broader population;
2. The study population consisted exclusively of soldiers and civilian military employees, which limits the applicability of the results to the general civilian population. The unique working conditions of this occupational group may significantly influence cardiovascular risk;
3. This study had a cross-sectional design, meaning that it presents only a single-time-point assessment of cardiovascular risk. It does not allow for causal inference or evaluation of changes in risk over time;
4. Although some lifestyle factors were considered, no detailed quantitative data were collected regarding dietary quality, caloric intake, or objectively measured physical activity (e.g., via accelerometers), which may limit the accuracy of lifestyle-related risk assessment;
5. Data on health behaviors and self-rated health status were based on participant self-reports, which may be subject to recall bias or social desirability bias—particularly in a hierarchical environment such as the military;
6. This study did not assess health-related motivation or interpersonal relationships, which may act as relevant psychosocial factors differentiating cardiovascular risk profiles between groups;
7. This analysis did not account for the menstrual cycle phase, which may influence the emotional and physiological state of female participants and affect certain risk factor assessments;
8. Metabolically heterogeneous phenotypes of obesity were not distinguished, which could have provided additional insights into cardiovascular risk beyond BMI-based categorization;
9. This study did not include an evaluation of occupational burnout, which may be a relevant stress-related factor contributing to cardiovascular risk, especially in demanding professional environments such as the military;
10. This analysis was limited to descriptive statistics, which may reduce the strength of the evidence and limit the ability to detect deeper associations between variables.

## Figures and Tables

**Table 1:** Characteristics of study participants according to quantitative risk factors for cardiovascular disease.

Variables	$\bar{x}$	SD	Me	Min	Max	Q1	Q3
systolic blood pressure	136.03	15.85	135.00	96.00	190.00	125.00	145.00
diastolic blood pressure	83.21	9.99	81.00	52.00	118.00	76.50	90.00
total cholesterol	222.16	44.85	216.40	117.00	459.60	193.70	250.90
LDL	135.92	37.64	134.50	35.00	353.60	111.30	160.10
HDL	61.36	18.25	59.00	31.00	199.00	49.00	72.00
non-HDL cholesterol	160.80	44.66	161.50	50.00	380.20	128.90	190.00
TG	141.42	91.53	114.55	13.00	608.00	82.00	171.50

**Table 2:** Occurrence of cardiovascular disease symptoms in the study participants by military service corps.

Variables		Military Service Corps [%]					
		Private Soldier	Non-Commissioned Officer	Junior Officer	Senior Officer	General, Admiral	Lack
chest pain	yes	17.39	7.87	12.07	2.63	-	9.43
	no	82.61	92.13	87.93	97.37	100.00	90.57
Statistics		Chi^2^ = 5.060, df = 5, *p* = 0.4085					
dyspnoea	yes	-	3.94	3.45	5.26	-	11.32
	no	100.00	96.06	96.55	94.74	100.00	88.68
Statistics		Chi^2^ = 6.467, df = 5, *p* = 0.2633					
dizziness	yes	13.04	5.51	3.45	5.26	-	13.21
	no	86.96	94.49	96.55	94.74	100.00	86.79
Statistics		Chi^2^ = 6.423, df = 5, *p* = 0.2671					
heart palpitations	yes	8.70	11.81	8.62	7.89	-	16.98
	no	91.30	88.19	91.38	92.11	100.00	83.02
Statistics		Chi^2^ = 3.142, df = 5, *p* = 0.6780					
pain in the lower limbs	yes	4.35	8.66	5.17	2.63	-	15.09
	no	95.65	91.34	94.83	97.37	100.00	84.91
Statistics		Chi^2^ = 6.533, df = 5, *p* = 0.2576					
headache	yes	30.43	29.13	18.97	31.58	-	41.51
	no	69.57	70.87	81.03	68.42	100.00	58.49
Statistics		Chi^2^ = 8.127, df = 5, *p* = 0.1493					
increased blood pressure	yes	17.39	18.11	17.24	36.84	-	13.21
	no	82.61	81.89	82.76	63.16	100.00	86.79
Statistics		Chi^2^ = 9.850, df = 5, *p* = 0.0795					

**Table 3:** Prevalence of cardiovascular disease risk factors by military service corps.

Variables		Military Service Corps [%]					
		Private Soldier	Non-Commissioned Officer	Junior Officer	Senior Officer	General, Admiral	Lack
overweight	yes	8.70	33.86	28.81	45.95	-	22.00
	no	91.30	66.14	71.19	54.05	100.00	78.00
Statistics		Chi^2^ = 12.260, df = 5, *p* = 0.0313					
obesity	yes	17.39	15.75	8.47	16.22	-	18.00
	no	82.61	84.25	91.53	83.78	100.00	82.00
Statistics		Chi2 = 2.721, df = 5, *p* = 0.7428					
incorrect diet	yes	13.04	42.52	38.98	43.24	-	38.00
	no	86.96	57.48	61.02	56.76	100.00	62.00
Statistics		Chi^2^ = 8.126, df = 5, *p* = 0.1493					
increased cholesterol levels	yes	8.70	17.32	11.86	24.32	-	22.00
	no	91.30	82.68	88.14	75.68	100.00	78.00
Statistics		Chi^2^ = 4.689, df = 5, *p* = 0.4548					
low physical activity	yes	26.09	18.11	11.86	21.62	-	38.00
	no	73.91	81.89	88.14	78.30	100.00	62.00
Statistics		Chi^2^ = 12.847, df = 5, *p* = 0.0248					
smoking	yes	26.09	22.05	15.25	32.43	-	16.00
	no	73.91	77.95	84.75	67.57	100.00	84.00
Statistics		Chi^2^ = 5.502, df = 5, *p* = 0.3576					
drinking alcohol	yes	13.04	19.69	15.25	18.92	-	4.00
	no	86.96	80.31	84.75	81.08	100.00	96.00
Statistics		Chi^2^ = 7.373, df = 5, *p* = 0.1942					
stress	yes	56.52	47.24	61.02	67.57	100.00	54.00
	no	43.48	52.76	38.98	32.43	-	46.00
Statistics		Chi^2^ = 7.477, df = 5, *p* = 0.1874					

**Table 4:** Self-assessment of health status of respondents, divided into military service corps (*p* < 0.0000).

Variables		Military Service Corps [%]					
		Private Soldier	Non-Commissioned Officer	Junior Officer	Senior Officer	General, Admiral	Lack
self-assessment of health	very good	30.43	22.83	54.24	18.92	-	14.00
	good	43.48	62.99	33.90	64.86	100.00	56.00
	sufficient	17.39	6.30	5.08	16.22	-	16.00
	wrong	4.35	-	-	-	-	-
	no opinion	4.35	7.87	6.78	-	-	14.00

**Table 5:** Self-assessment of respondents’ knowledge about cardiovascular diseases by military service corps (*p* = 0.4102).

Variables		Military Service Corps [%]					
		Private Soldier	Non-Commissioned Officer	Junior Officer	Senior Officer	General, Admiral	Lack
self-assessment of knowledge	very good	-	0.79	3.39	5.26	-	4.00
	good	22.73	27.78	18.64	21.05	100.00	30.00
	sufficient	63.64	53.97	64.41	65.79	-	42.00
	below sufficient	4.55	11.11	10.17	7.89	-	8.00
	no opinion	9.09	6.35	3.39	-	-	16.00

**Table 6:** Readiness of respondents to increase knowledge about cardiovascular diseases (*p* = 0.5574).

Variables		Military Service Corps [%]					
		Private Soldier	Non-Commissioned Officer	Junior Officer	Senior Officer	General, Admiral	Lack
readiness to increase knowledge	yes	81.82	64.29	66.10	78.95	100.00	62.75
	no	-	9.52	8.47	7.89	-	5.88
	no opinion	18.18	26.19	25.42	13.16	-	31.37

## Data Availability

Data are contained within this article.
